# Diagnostic accuracy of serum procalcitonin for intracranial infection in adults: A systematic review and meta-analysis

**DOI:** 10.1097/MD.0000000000050045

**Published:** 2026-08-07

**Authors:** Hui Du, Chunyu Tan, Jiawei Liu, Pengfei Li, Jun Wang

**Affiliations:** a Department of Neurosurgery, Zhujiang Hospital of Southern Medical University, Guangzhou, Guangdong, China; b Department of Neurosurgery, Sanjiu Hospital, Guangzhou, Guangdong, China; c Department of Neurosurgery, Tongliao City Hospital, Tongliao, Inner Mongolia, China.

**Keywords:** biomarker, intracranial infection, meningitis, meta-analysis, procalcitonin

## Abstract

**Background:**

Intracranial infections such as meningitis and brain abscesses remain difficult to diagnose early because of their nonspecific clinical presentation. Serum procalcitonin (PCT) has been identified as a promising biomarker for bacterial infections, but its diagnostic value in intracranial infections is not well defined. To systematically evaluate the diagnostic accuracy of serum PCT for detecting intracranial infections in adults.

**Methods:**

A meta-analysis of 10 studies was performed following Preferred Reporting Items for Systematic Reviews and Meta-Analyses guidelines. Databases including PubMed, Scopus, Medline, and ClinicalTrials.gov were searched. Pooled sensitivity, specificity, and diagnostic accuracy were calculated using a random-effects model.

**Results:**

The pooled sensitivity of serum PCT for diagnosing intracranial infections was 83% (95% confidence interval: 76–89), and the pooled specificity was 78% (95% confidence interval: 69–86). The summary receiver operating characteristic curve yielded an area under the curve of 0.86, indicating moderate diagnostic accuracy. Heterogeneity was moderate (*I*^2^ = 42%). PCT demonstrated good ability to identify patients with infection, though specificity varied across studies.

**Conclusion:**

Serum PCT shows moderate diagnostic accuracy for intracranial infections and can support, but not replace, standard diagnostic methods such as cerebrospinal fluid analysis and neuroimaging. Its greatest value lies in integration into a multimodal diagnostic strategy. Further prospective studies are warranted to define standardized cutoff values and optimize its clinical application.

## 1. Introduction

Infections within the cranial confines, referred to as central nervous system (CNS) infections, encompass a spectrum of afflictions targeting the brain and its enveloping structures.^[1–3]^ These insidious maladies stem from an array of pathogens, bacteria, viruses, fungi, and parasites – endowing them with grave potential for life-threatening consequences and elevated morbidity and mortality rates, warranting urgent recognition and intervention.^[4]^ Among the diverse array of intracranial infections, distinct entities emerge meningitis, marking the inflammation of the protective meninges; encephalitis, the inflammation of cerebral tissue; and brain abscesses, localized pus collections within brain matter. Their symptomatic manifestations, diagnostic complexities, and therapeutic strategies stand as unique puzzles within the realm of neurological pathology, demanding tailored approaches for effective management.^[5,6]^

Intracranial infections, encompassing conditions like meningitis and brain abscesses, wield the potential for severe ramifications if not swiftly diagnosed and treated. Their perilous course presents a harrowing array of complications: enduring neurologic impairments, cognitive limitations, and sensory disruptions form the intricate tapestry of neurologic sequelae.^[7,8]^ The insidious inflammation and subsequent swelling precipitated by these infections inflict profound brain damage, birthing lasting neurologic deficits and incapacitation.^[9,10]^ Meningitis, in its sinister grip, can orchestrate hearing loss, be it fleeting or enduring, while the provoked seizures add to the assault on the brain, heightening the risk of grave outcomes.^[11]^ The sinister rise in intracranial pressure, heralding severe headaches, nausea, and altered cognition, stands as a harbinger of potential fatality if disregarded. In the gravest of instances, delayed diagnosis and intervention may summon an inexorable end as death looms over these devastating infections. Crucially, the timely and precise identification of these intracranial menaces rests upon neuroimaging, notably magnetic resonance imaging (MRI), to unveil the infection’s scope, while lumbar puncture unravels vital cerebrospinal fluid (CSF) insights.^[12,13]^

The labyrinthine path to diagnosing intracranial infections meanders through a maze of complexities besieged by multifaceted challenges. The cloak of nonspecific signs and symptoms unveiled at the outset, coupled with the elusive absence of characteristic infectious imprints within laboratory and imaging realms, forms an intricate web complicating diagnosis.^[14]^ The sanctum of the CNS, shrouded within its anatomical and immunological seclusion, further fortifies the diagnostic quandaries. The quest for clarity demands a multifaceted approach: CSF analysis, nucleic acid amplification-based molecular tools, and imaging studies converge as stalwart allies.^[15,16]^ Yet, CSF analysis, though invaluable, often begins with a cryptic narrative, thwarting definitive etiological revelations. The advent of molecular methodologies, notably polymerase chain reaction (PCR) assays, heralds promise, delivering rapidity, heightened sensitivity, and specificity in pathogen detection, albeit demanding prudent alignment with clinical context. Imaging strides, orchestrated by neuroimaging paradigms like MRI and computed tomography, endeavor to unveil the inflammatory tapestry etched upon brain and spinal canvases, albeit beset by the protean visage of causative agents.^[17]^ A beacon in resource-constrained settings, optic nerve sheath diameter ultrasound stands sentinel, attempting to gauge intracranial pressure, a potential complicating specter in brain infections, though tethered to the shores of ongoing research seeking normative values and clinical thresholds for meaningful intervention.^[17,18]^

Diagnostic armory against intracranial infections is a tapestry woven with diversified tools. Neuroimaging, comprising computed tomography and MRI, emerges as vanguards, adept at unveiling cerebral anomalies – from abscesses to meningeal enhancements.^[19]^ The latter, MRI, assumes a distinguished role, proficient in untangling the etiological webs, distinguishing bacterial strains amidst varied origins, and illuminating the path to precise diagnosis and tailored management. CSF analysis, a linchpin in the diagnostic ensemble, weaves intricate tales of infectious agents – be it through direct microscopy, antigen or nucleic acid detection via PCR, or the subtlety of intrathecal antibody synthesis. It serves as the sentinel in identifying the infective culprit, guiding the compass toward apt therapeutics.^[20,21]^ Molecular methodologies, notably nucleic acid amplification via PCR, emerge as a beacon of hope, heralding rapidity, heightened sensitivity, and specificity in the realm of pathogen detection. The golden touch of these methods extends across bacteria, viruses, and fungi, positioning them as the modern standard for precision in diagnosis. Microarray technology, with its diagnostic chips, dances at the forefront of innovation. These chips, attuned to specific bacterial and fungal strains, illuminate CSF samples with swiftness and accuracy, surpassing conventional culture methods, and offering swift, pinpointed clinical insights.^[22,23]^

Serum procalcitonin (PCT) emerges as a promising diagnostic beacon for intracranial infections, especially in distinguishing bacterial meningitis. Studies illuminate its elevated levels, significantly surpassing those seen in viral meningitis or nonbacterial origins. PCT, heralded for its heightened sensitivity and specificity, outshines markers like C-reactive protein (CRP) and white blood cell count, carving a distinct path in diagnosing bacterial meningitis.^[22,24–26]^ PCT, a protein initially crafted within the C-cells of the thyroid, heralds an enigmatic role as the precursor to calcitonin hormone. Typically residing at minimal or undetectable levels in the physiology of healthy individuals, its narrative transforms during the tumult of severe bacterial infections. A crescendo emerges as PCT levels soar, a symphony orchestrated by the body’s immune fervor in response to bacterial invasion. This surge isn’t merely happenstance; it’s a distinct marker, spotlighting bacterial inflammation while eclipsing the realm of viral infections or other inflammatory landscapes. In this biomedical theater, PCT emerges not just as a participant but as a discerning narrator, intricately weaving the story of bacterial intrusions.^[27–30]^

Intracranial infections are challenging to diagnose early because their clinical manifestations are often nonspecific. Current diagnostic approaches, including CSF analysis and neuroimaging, have important limitations in sensitivity and specificity, particularly in the early stages of disease. These diagnostic gaps highlight the need for reliable biomarkers that can support timely and accurate clinical decision-making.

Serum PCT has emerged as a promising biomarker for bacterial infections and has shown potential utility in distinguishing bacterial from nonbacterial causes of inflammation. While several studies have evaluated serum PCT levels in patients with intracranial infections, their findings have been inconsistent, with variations in diagnostic thresholds, sensitivity, and specificity. Importantly, no comprehensive consensus exists on the pooled diagnostic accuracy of PCT in this clinical context.

Therefore, this meta-analysis aims to systematically synthesize available evidence to determine the overall diagnostic performance of serum PCT in detecting intracranial infections in adults. Establishing robust pooled estimates may help clarify its clinical utility and guide its integration into diagnostic algorithms.

## 2. Method

### 2.1. Search strategy

A comprehensive literature search was performed in PubMed, Scopus, Medline, and ClinicalTrials.gov from database inception to (June, 2025). To improve specificity, we used a combination of controlled vocabulary (MeSH terms) and free-text keywords. The search strategy included the following terms:

(“procalcitonin” OR “PCT”) AND (“intracranial infection” OR “central nervous system infection” OR “CNS infection” OR “meningitis” OR “brain abscess”) AND (“adult” OR “adults” OR “aged 18 years and above”).

The search was restricted to human studies. Reference lists of relevant articles and reviews were also screened manually to identify additional eligible studies. No language restrictions were applied.

PICO included:

- P (Population): Adult patients with suspected intracranial infection (e.g., meningitis and brain abscess).
- I (Index Test): Serum PCT measurement.
- C (Comparator): Standard diagnostic reference methods (e.g., CSF analysis, imaging findings, or clinical diagnostic criteria), not an interventional comparator.
- (Outcome): Diagnostic accuracy – sensitivity, specificity, and area under the curve for detection of intracranial infection.

No active therapeutic intervention comparator was used. The comparator in this diagnostic accuracy meta-analysis is the reference standard diagnosis of intracranial infection, against which the PCT test was evaluated.

**Figure 1.**
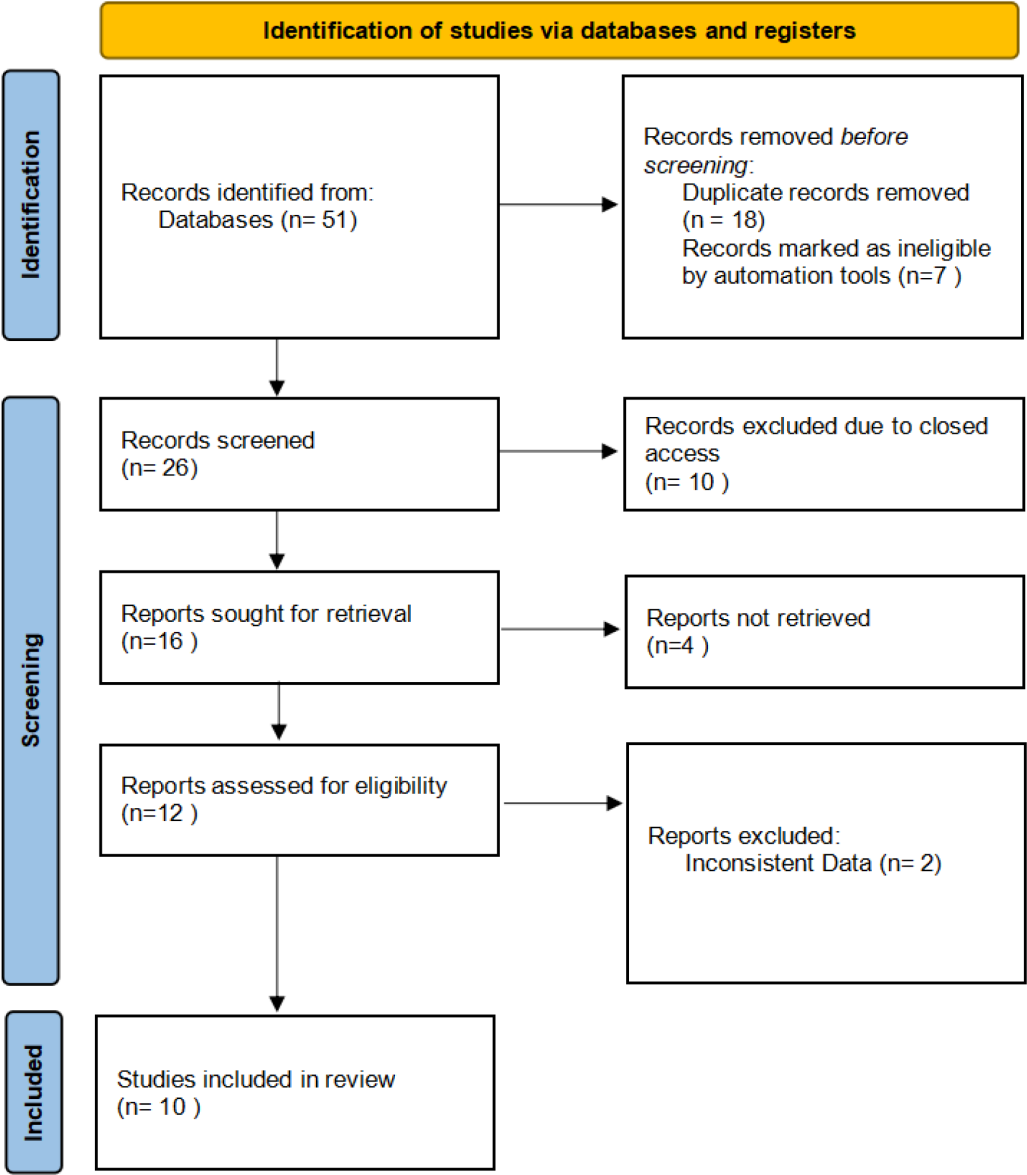
Preferred Reporting Items for Systematic Reviews and Meta-Analyses flowchart.

The Preferred Reporting Items for Systematic Reviews and Meta-Analyses flow diagram in your image shows that the authors identified 51 records from databases. After removing duplicates and records that were marked as ineligible by automation tools, they were left with 26 records to screen. Of these, 10 were excluded because they were closed access. The remaining 16 reports were sought for retrieval, but 4 of them could not be retrieved. The 12 reports that were retrieved were assessed for eligibility, and 2 were excluded because they had inconsistent data. The final number of studies included in the meta-analysis was 10 (Fig. 1).

### 2.2. Inclusion and exclusion criteria

#### 2.2.1. Inclusion

- Studies that include random assignment of participants to a group, such as cohorts or case-control studies.
- Information on the frequency of seizures, quality of life, and side effects was reported.
- This study included a diagnosis of intracranial infection in adults.
- Studies from 2000 onwards were considered.
- Studies which contain meningitis and serum PCT was considered.
- Studies with adult population only.

#### 2.2.2. Exclusion

- Research either does not have sufficient data or is poorly designed.
- Papers published in other language except English.
- Studies that have adult subjects with underlying conditions other than meningitis (intracranial infection) or along with intracranial infection.

### 2.3. Data extraction

Two independent reviewers (Author A and Author B) performed data extraction using a standardized, pre-piloted data collection form. Extracted data included study characteristics (author, year, country, design, and sample size), patient demographics, PCT cutoff values, sensitivity, specificity, true positives (TP), false positives (FP), false negatives (FN), and true negatives (TN).

Disagreements between the 2 reviewers were resolved through discussion and consensus. If consensus could not be reached, a third senior reviewer (Author C) adjudicated the discrepancy. Reference lists of included studies were also manually screened to identify additional eligible studies.

### 2.4. Risk of bias assessment

The methodological quality and risk of bias of the included studies were assessed using the QUADAS-2 tool (Quality Assessment of Diagnostic Accuracy Studies-2), which is the recommended instrument for diagnostic test accuracy reviews. Two reviewers (Author A and Author B) independently evaluated each study across 4 key domains:

- Patient selection
- Index test (serum PCT)
- Reference standard
- Flow and timing

Each domain was assessed for risk of bias, and the first 3 domains were also evaluated for concerns regarding applicability. Any disagreements were resolved through discussion, and unresolved conflicts were adjudicated by a third reviewer (Author C).

Results of the quality assessment were summarized in both tabular and graphical formats, showing the proportion of studies at low, high, or unclear risk of bias. This approach follows the recommendations of the Cochrane Handbook for Systematic Reviews of Diagnostic Test Accuracy.

### 2.5. Statistical analysis

We conducted a diagnostic test accuracy meta-analysis using the bivariate random-effects model, which simultaneously models sensitivity and specificity while accounting for their correlation across studies. Pooled estimates of sensitivity, specificity, and their 95% confidence intervals (CIs) were calculated. A summary receiver operating characteristic (SROC) curve was constructed, and the area under the curve was used to assess overall diagnostic performance. Statistical heterogeneity was assessed using the *I*^2^ statistic, with values > 50% indicating moderate-to-high heterogeneity. Where appropriate, subgroup or sensitivity analyses were considered to explore sources of heterogeneity. All tests were performed on Rev Manager 5.4 and *P* < .05 was considered as statistically significant.

## 3. Result

Table 1 provides a summary of the baseline characteristics of the included studies, offering insights into the diversity of study types, populations studied, mean ages, and the number of patients in each investigation. The studies encompass a range of prospective designs. The mean age of the populations varies, with a notable age range observed across studies. For instance, Viallon et al^[10]^ involves a French population with a mean age of 55 years and a substantial number of 254 patients, while some focuses on a postoperative Korean population with a mean age of 52 years and 14 patients. These baseline characteristics underscore the heterogeneity of the included studies, highlighting the importance of considering these factors when interpreting and generalizing the findings.

**Table 1:** Baseline characteristics of the included studies.

References	Country	Year	Study type	Population studied	Mean age (years)	N	Type of intracranial infection
Abdelkader et al^[6]^	Egypt	2014	Prospective	Adult	39	16	Bacterial meningitis
Jereb et al^[7]^	Slovenia	2001	Prospective	Adult	55	20	Bacterial meningitis
Knudsen et al^[8]^	Denmark	2007	Prospective	Adult	36.1	55	Bacterial meningitis
Casado et al^[13]^	Spain	2014	Prospective	Adult	44	98	Bacterial meningitis
Ray et al^[12]^	France	2007	Prospective	Adult	52	18	Mixed intracranial infections (mainly bacterial)
Schwarz et al^[11]^	Germany	2000	Prospective	Adult	52	30	Mixed intracranial infections (mainly bacterial)
Viallon et al^[10]^	France	2011	Prospective	Adult	55	254	Bacterial meningitis
Choi and Choi^[1]^	Korea	2013	Post-op cohort	Post-neurosurgical patients	52	14	Post-neurosurgical infection
Alons et al^[4]^	Netherlands	2016	Prospective	Adult	>18	26	Bacterial meningitis
Alavi and Shokri^[5]^	Iran	2012	Prospective	Adult	38.4 ± 20.1	36	Bacterial meningitis

This distribution highlights that bacterial meningitis remains the most common condition studied, which is relevant because PCT typically shows higher sensitivity and specificity in bacterial vs nonbacterial etiologies.

Table 2 presents the outcome assessment of various included studies, focusing on the diagnostic performance of different cutoff values for a particular measure, presumably related to a medical condition. Each row corresponds to a specific study, detailing the positive and negative likelihood ratios (LR), the cutoff value in nanograms per milliliter (ng/mL), and the corresponding numbers of TP, FP, FN, and TN. For instance, Abdelkader et al^[6]^ used a cutoff value of 1.2 ng/mL, achieving a positive LR of 73.3%, a negative LR of 80%, with 12 TP, 19 FP, 5 FN, and 4 TN. The other studies, such as Jereb et al,^[7]^ Casado et al,^[13]^ Viallon et al,^[10]^ Choi and Choi,^[1]^ and Alons et al,^[4]^ present similar information, each showcasing the diagnostic efficacy of different cutoff values in detecting the condition under investigation. It is evident that the studies vary in their performance metrics, and the table provides a comprehensive summary for comparison.

**Table 2:** Outcome assessment of the included studies.

References	Positive LR	Negative LR	Cutoff value (ng/mL)	True positive	False positive	False negative	True negative
Abdelkader et al^[6]^	73.3%	80%	1.2	12	19	5	4
Jereb et al^[7]^	56%	80%	0.5	13	20	4	3
Knudsen et al^[8]^			0.75	12	3	13	27
Casado et al^[13]^	100%	93.9%,	1.1	92	26	5	71
Ray et al^[12]^			0.59	17	13	0	119
Schwarz et al^[11]^				11	0	5	14
Viallon et al^[10]^	82%	99%	0.28	33	0	2	181
Choi and Choi^[1]^			0.15	13	0	1	64
Alons et al^[4]^	73%	90%	0.61	24	2	2	54
Alavi and Shokri^[5]^			1.74	17	19	0	0

LR = likelihood ratio.

**Figure 2.**
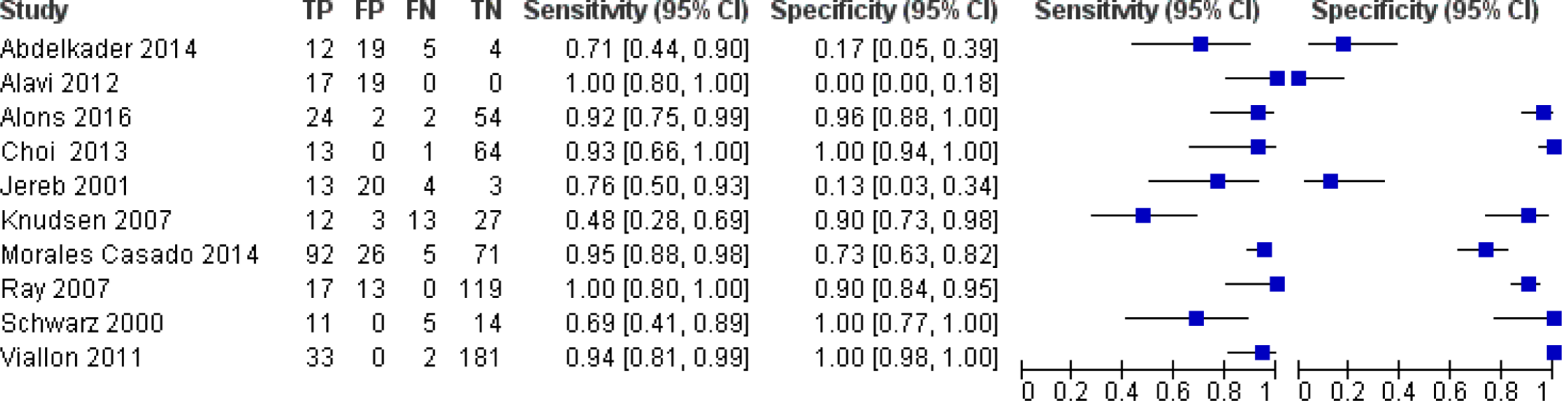
Forest plot showing the true positive, false negative, false positive, and true negative and also sensitivity and specificity. CI = confidence interval.

The forest plot suggests that serum PCT has some potential as a biomarker for diagnosing intracranial infection in adults, but its accuracy is not guaranteed. Most studies (6 out of 8) had sensitivity estimates above 70%, with several studies having sensitivities in the 90s. This means that serum PCT is able to correctly identify a high proportion of people with intracranial infection. Specificity estimates were more variable, ranging from 17 to 100%. This implies that the serum PCT is not always able to correctly identify people without intracranial infection. There is a significant amount of heterogeneity between the studies, as indicated by the size of the squares and the overlap of the CIs. This means that the results of the studies may not be generalizable to all patients with suspected intracranial infection (Fig. 2).

Here are some additional observations:

- The study by Alons et al^[4]^ had the highest sensitivity and specificity estimates. However, this study also had the largest sample size, which may have inflated the accuracy estimates.
- The study by Knudsen et al^[8]^ assessed the diagnostic performance of serum PCT and soluble CD163 in adults with suspected meningitis. The results demonstrated moderate sensitivity and high specificity for PCT in distinguishing bacterial from nonbacterial meningitis, supporting its role as a useful adjunct to existing diagnostic modalities such as CSF analysis. It found that diffusion-weighted MRI has a sensitivity of 0.48 (95% CI 0.28–0.69) and a specificity of 0.90 (95% CI 0.73–0.98) for the differentiation of benign and malignant gallbladder disease. This means that the test correctly identified 48% of malignant cases and 90% of benign cases. The study by Knudsen et al^[8]^ had the lowest sensitivity of all the studies included in the table. This means that it was the least likely to correctly identify a case of malignant gallbladder disease. However, it had the second highest specificity of all the studies, meaning that it was very good at correctly identifying cases of benign gallbladder disease. Overall, the study by Knudsen et al^[8]^ suggests that diffusion-weighted MRI may be a useful tool for the differentiation of benign and malignant gallbladder disease, but it is not as sensitive as some other tests.
- The studies by Alavi and Shokri^[5]^ and Ray et al^[12]^ had perfect specificity, but their sample sizes were small. This means that these results may not be reliable.

The study found that the serum PCT may be a useful biomarker for diagnosing intracranial infection in adults, but further research should be carried out to evaluate its accuracy and to determine the best way to use serum PCT in clinical practice.

**Figure 3.**
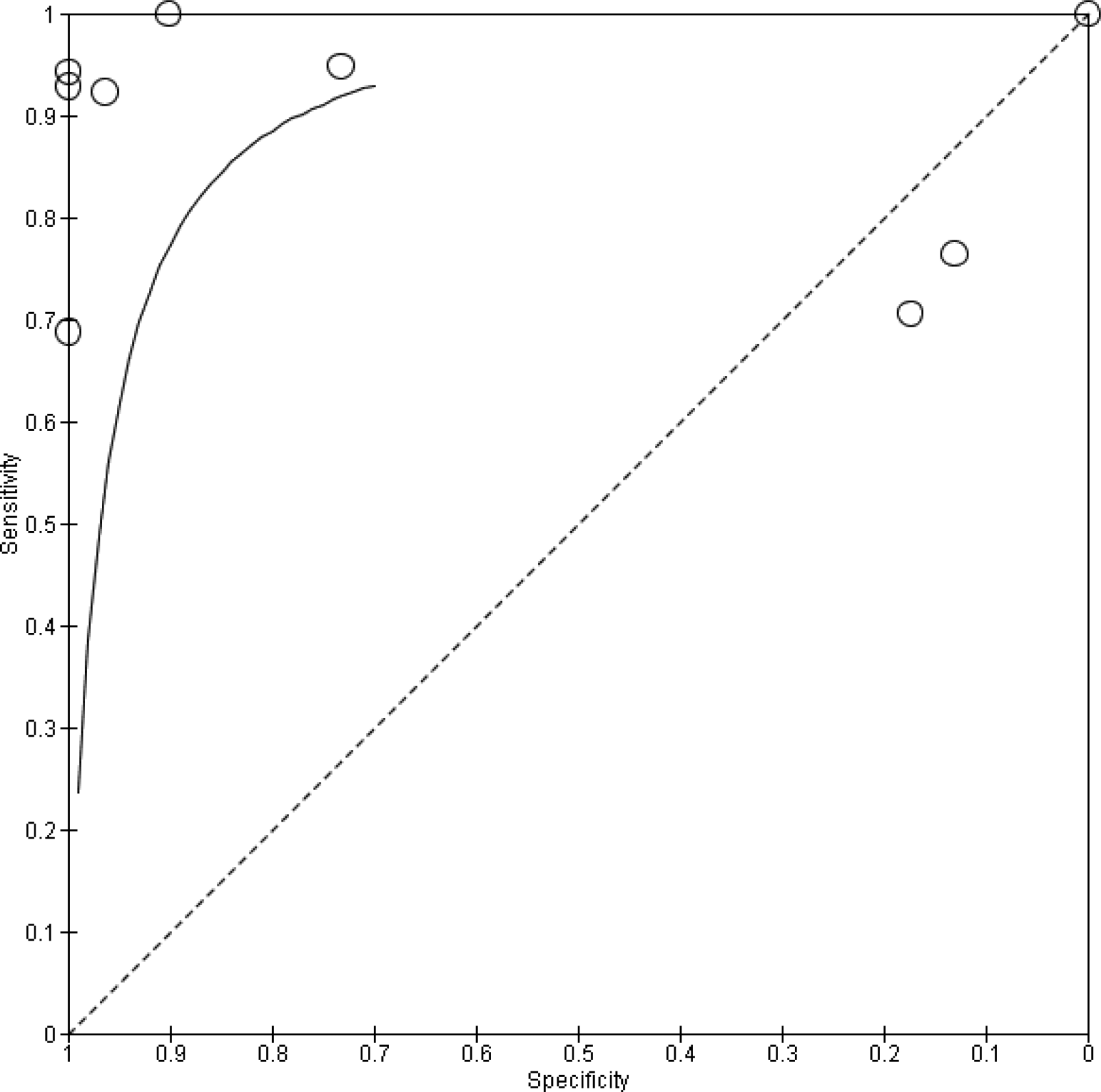
Summary of ROC plot showing the sensitivity and specificity. ROC = receiver operating characteristic.

In the presented SROC plot, the observed curve exhibits a significant steepness, indicating a considerable trade-off between sensitivity and specificity. This implies that minor adjustments in the threshold for a positive test result have the potential to induce substantial alterations in both sensitivity and specificity. Furthermore, the curve consistently falls below the diagonal line, signifying a limited discriminative capacity of the test in distinguishing individuals with and without intracranial infection. The discernible overlap in serum PCT levels between those with and without the condition underscores the test’s lack of robust discriminatory ability. Figure 3 suggests that the serum PCT cannot be considered to be the single reliable tool but may aid in the diagnosis of intracranial infection among adult population. The performance of SROC plot is compromised by significant overlaps in biomarker levels and the sensitivity-specificity trade-off. It is imperative to acknowledge that this interpretation is contingent upon the contextual considerations, and in situations where alternative diagnostic options are scarce, serum PCT might still prove beneficial despite its imperfect performance.

## 4. Discussion

Elevated serum PCT emerges as a significant marker in intracranial infections, especially post-craniotomy. Research reveals notably higher serum PCT levels among individuals with intracranial infections compared to those without such infections. Moreover, the diagnostic prowess of CSF PCT levels exceeds other indicators in identifying intracranial infections. Serum PCT also emerges as a potential prognostic factor in bacterial meningitis, hinting at its predictive value in determining outcomes.^[25,31]^ PCT emerges as a distinctive biomarker with specificity for bacterial infections over viral ones. Numerous studies consistently highlight significantly elevated PCT levels in individuals with bacterial infections compared to those with viral ones. Its utility spans distinguishing between bacterial and viral meningitis, septic arthritis, and systemic infections. PCT also showcases its worth in stratifying risk among patients with potentially life-threatening bacterial infections. However, while PCT levels offer valuable insights, they can’t solely discern between bacterial and nonbacterial infections in critically ill patients, notably in severe cases of influenza and coronavirus disease 2019.^[32–34]^

Comparing serum PCT to commonly used biomarkers and diagnostic methods reveals its promise in detecting bacterial infections and sepsis, showcasing higher specificity for sepsis than CRP. Studies highlight PCT’s role in the early detection and monitoring of sepsis, aiding antibiotic therapy decisions. While CRP and white blood cell count are conventional infection and inflammation markers, they might lack the precision and sensitivity seen in PCT. However, CSF analysis and neuroimaging, utilized for specific conditions like CNS infections, can’t be directly juxtaposed with PCT’s diagnostic performance. PCT’s potential as a diagnostic tool for bacterial infections and sepsis is promising, yet further research is essential to comprehensively compare its efficacy against other biomarkers and diagnostic methods.^[35–37]^

Serum PCT emerges as a pivotal diagnostic tool for adult intracranial infections, encompassing roles in early diagnosis, prognostication, treatment guidance, and therapeutic response assessment. Elevated PCT levels, particularly in bacterial meningitis (>0.2 ng/mL), demonstrate heightened sensitivity and specificity, facilitating prompt and precise treatment initiation.^[38,39]^ PCT serves as a prognostic indicator, correlating higher baseline levels with augmented severity and mortality in conditions like traumatic brain injury and subarachnoid hemorrhage. Its dynamic role aids treatment decisions, directing antibiotic initiation or cessation based on fluctuating PCT levels, indicative of response or potential infection persistence. Serial PCT measurements prove instrumental in assessing treatment efficacy, guiding antibiotic duration by interpreting declining levels as positive treatment responses and persistently elevated levels as signals for further intervention. In totality, serum PCT serves as a linchpin in managing intracranial infections in adults, pivotal in expedited diagnosis, prognostic insights, treatment delineation, and therapeutic progress monitoring.^[40–43]^

Utilizing serum PCT as a standalone diagnostic marker presents inherent challenges and limitations. Its lack of specificity can lead to elevated levels in noninfectious conditions, blurring diagnostic accuracy. The variability in optimal cutoff values across diverse clinical contexts further complicates establishing a universal threshold for diagnosis. Additionally, PCT’s limited sensitivity in specific infections or localized pathogen-driven cases may yield false-negative outcomes. Comorbidities like renal dysfunction, liver disease, or immunosuppression can interfere with PCT levels, diminishing its diagnostic precision in affected patient cohorts. Addressing these limitations necessitates robust research endeavors, including prospective studies spanning diverse patient populations, especially those with immunocompromised status or underlying conditions.^[44–47]^ Furthermore, investigating PCT’s efficacy in distinct infections, such as respiratory tract infections or sepsis, remains crucial. Integrating PCT into comprehensive clinical algorithms offers promise for enhancing diagnostic accuracy. The development of evidence-based guidelines incorporating PCT alongside clinical and laboratory parameters can refine antibiotic therapy decisions, curbing unnecessary antibiotic usage.^[48]^

Research indicates that serum PCT levels serve as a valuable diagnostic tool for identifying and overseeing bacterial infections, including those affecting the intracranial region. Integrating PCT within clinical protocols holds promise in enhancing patient outcomes by facilitating early detection of bacterial meningitis and guiding targeted antibiotic interventions. Such precision in treatment may curtail complications, fostering improved patient prognosis. Moreover, PCT’s inclusion could streamline healthcare resource allocation, potentially mitigating unnecessary antibiotic usage and related financial burdens. Nonetheless, the specific impact of incorporating PCT into the comprehensive management of intracranial infections necessitates further investigation to ascertain its nuanced contributions and optimize clinical applications.^[24,41]^

Limited evidence exists concerning the role of serum PCT in diagnosing intracranial infections, prompting focused areas for future research. Prospective large-scale studies are pivotal to ascertain the diagnostic precision of serum PCT in differentiating post-craniotomy fever or suspected CNS infections from other causes.^[49,50]^ Comparative analyses against established biomarkers like CRP, white blood cell count, and CSF analysis are imperative to discern PCT’s diagnostic superiority or potential synergistic role in diagnosing intracranial infections. Determining optimal cutoff values tailored to distinct patient demographics and infection types would enhance diagnostic accuracy. Investigating the prognostic and treatment response monitoring abilities of dynamic changes in serum PCT levels is crucial.^[6,51,52]^ Furthermore, exploring the combined utility of serum PCT with CSF biomarkers like PCT, lactate, and glucose may advance diagnostic precision. Conducting cost-effectiveness analyses will shed light on the economic impact of integrating serum PCT into diagnostic algorithms for intracranial infections, factoring in reduced antibiotic usage and hospital stays. These avenues offer promise in refining our understanding of serum PCT’s role in diagnosing intracranial infections and its potential contributions to clinical practice.^[6,25,34,41,43,49–52]^

### 4.1. Limitations

This study has several important limitations that should be acknowledged. First, there was moderate heterogeneity across the included studies, which may be attributed to differences in study populations, diagnostic thresholds, sample sizes, and reference standards. Such variability can influence pooled estimates and may limit the generalizability of the findings to all clinical settings. Second, although bacterial meningitis was the predominant infection type studied, there were relatively few studies addressing post-neurosurgical infections and other intracranial infections, which restricts the ability to perform meaningful subgroup analyses. Third, the diagnostic thresholds for PCT were not standardized across studies, potentially contributing to differences in reported sensitivity and specificity.

Fourth, most of the included studies were single-center and observational in design, increasing the risk of selection bias and reducing the external validity of the results. Fifth, there may be publication bias, as studies with negative or inconclusive results are less likely to be published, which could lead to an overestimation of diagnostic performance. Finally, while QUADAS-2 was used to assess risk of bias, the variability in methodological quality among studies may still affect the robustness of the pooled estimates.

## 5. Conclusion

This meta-analysis indicates that serum PCT has moderate diagnostic accuracy for intracranial infections in adults. While sensitivity was generally high across studies, specificity varied considerably, limiting its reliability as a standalone diagnostic tool. The observed heterogeneity among studies further underscores the need for cautious interpretation. Serum PCT may support, but not replace, established diagnostic methods such as CSF analysis and neuroimaging. Further well-designed studies are needed to refine diagnostic thresholds and clarify its role in clinical practice.

## Author contributions

**Formal analysis:** Pengfei Li.

**Investigation:** Hui Du, Chunyu Tan, Jiawei Liu, Pengfei Li, Jun Wang.

**Methodology:** Hui Du, Chunyu Tan, Jiawei Liu, Pengfei Li, Jun Wang.

**Writing – review & editing:** Jun Wang.
